# Emory Wisconsin Card Sorting Test and Trail Making Test B as Measures of Executive Dysfunction

**DOI:** 10.1002/alz.095677

**Published:** 2025-01-09

**Authors:** Gabrielle A Lincoln, Subhamoy Pal, Jonathan M Reader, Savannah G Rose, Amanda Cook Maher, Bruno Giordani

**Affiliations:** ^1^ University of Michigan, Ann Arbor, MI USA; ^2^ Michigan Alzheimer’s Disease Research Center, Ann Arbor, MI USA; ^3^ University of Michigan Medical School, Ann Arbor, MI USA

## Abstract

**Background:**

Wisconsin Card Sorting Test (WCST) and Trail Making Test‐Part B (Trails B) are commonly used neuropsychological measures of executive functioning. Previously, Trails B was found to be more sensitive than the WCST in predicting executive dysfunction. The Emory WCST (eWCST) was adapted to remove cards with multiple sort options and duplicate cards, creating a more direct measure of executive function. This study aimed to assess whether the eWCST was a better predictor of executive dysfunction compared to Trails B.

**Method:**

Data from 864 participants enrolled in the longitudinal University of Michigan Memory and Aging Project within the Michigan Alzheimer’s Disease Center were analyzed (34.3% Male, 34.7% Black/African American). Participants completed baseline neuropsychological testing and other Unified Data Set activities and received a consensus diagnosis: Cognitively Normal, amnestic Mild Cognitive Impairment (aMCI), non‐amnestic MCI (naMCI), or dementia due to suspected Alzheimer’s disease (AD). eWCST total errors and Trails B completion time were standardized and converted to T‐scores. Mean test scores within each diagnostic group were compared via Welch 2 sample t‐tests. Analysis of variance (ANOVAs) were used to compare group differences in eWCST and Trails B performance. Multinomial logistic regression was used to predict cognitive diagnosis using eWCST and Trails B performance.

**Result:**

Within the three impaired groups (aMCI, naMCI, AD), mean T‐scores were lower (e.g., less impaired) for eWCST compared to Trails B (*p*’s<0.01). There was no between‐test difference for the cognitively normal group. All four groups showed significant differences for Trails B scores while eWCST differences were seen between all groups except between aMCI and naMCI. Logistic regression results found Trails B performance distinguished AD from both MCI groups (aMCI OR = 0.97, *p*<0.001; naMCI OR = 0.98, *p*<0.01), whereas eWCST errors trended toward differentiating AD from aMCI (*p* = 0.06) but not naMCI.

**Conclusion:**

Findings suggest Trails B completion time better differentiates AD from both amnestic and non‐amnestic MCI compared to the eWCST, similar to previous findings (Hammers et al., 2015). Determining the best predictors of cognitive diagnosis enables the selection of a concise testing battery to minimize participant burden and maximizing diagnostic utility, given the limited time and resources available at research centers.

